# Hearing loss in congenital Zika virus^☆^

**DOI:** 10.1016/j.bjorl.2016.08.007

**Published:** 2016-09-01

**Authors:** Viroj Wiwanitkit

**Affiliations:** Surindra Rajabhat University, Institute of Natural Medicine Science Development and Establishment Project, Suvannhabhumi Clinical Training, Research and Development Center, Surin Province, Thailand

Dear Editor,

The recent article on “*Hearing loss in congenital Zika virus*” is very interesting.^1^ Leal et al. reported an interesting case of sensorineural hearing loss in a case of congenital Zika virus.^1^ In fact, it is no doubt that Zika virus can induce teratogenic effect and the main pathology is on the fetal neurological system. However, the myth is the exact mechanism underlying the hearing loss in the present case. In fact, the direct viral invasion is believe to be the main pathogenesis of neurological defect in congenital Zika virus infection.^2^ In the present report, the interesting concern is the non-fatal brain involvement but neuro-auditory system defect. Generally, the brain pathology in congenital Zika virus infection is at cortex^3^ and not common at brain stem, hence, it should rarely involve the neuro-auditory system. Indeed, there are many viral infections that can result in congenital neurosensory hearing loss such as Cytomegalovirus (CMV) infection.^3^ There are many possible explanation for the present interesting case. In the present case, since there is no clear evidence when the mother got infection, the Zika virus intra-utero infection might occur after the brain development. There might also be prior silent concomitant infection that can already involve auditory system and induce neurosensory hearing loss (such as CMV infection, which can be silent in the pregnant).^4^

## Conflicts of interest

The author declares no conflicts of interest.

## References

[1] Leal M.C., Muniz L.F., Caldas Neto S.D., van der Linden V., Ramos R.C. (2016). Sensorineural hearing loss in a case of congenital Zika virus. Braz J Otorhinolaryngol.

[2] Wiwanitkit V. (2016). Placenta, Zika virus infection and fetal brain abnormality. Am J Reprod Immunol.

[3] Mlakar J., Korva M., Tul N., Popović M., Poljšak-Prijatelj M., Mraz J. (2016). Zika virus associated with microcephaly. N Engl J Med.

[4] Calvani M. (1991). Asymptomatic congenital cytomegalovirus infection. Minerva Pediatr.

